# Supplementary material for: RIP5 Interacts with REL1 and Negatively Regulates Drought Tolerance in Rice
Source: Cells. 2024 May 21;13(11):887. doi: 10.3390/cells13110887 (PMC11172185; doi:10.3390/cells13110887)
Supplement: Supplementary file 1 [file cells-13-00887-s001.zip › cells-3002690-supplementary.pdf]

Supplementary Materials:

**Oryza sativa Japonica Group DNA, chromosome 6, cultivar: Nipponbare, complete sequence**

Sequence ID: AP014962.1 Length: 31248787 Number of Matches: 2

Range 1: 21954299 to 21954934 GenBank Graphics ▼ Next Match ▲ Previous Match

| Score | Expect | Identities | Gaps | Strand |
|---|---|---|---|---|
| 1175 bits(636) | 0.0 | 636/636(100%) | 0/636(0%) | Plus/Plus |

Features: os06g0567900
os06g0567950

```
Query  1         GGGCGCTACAGGTGGTCCATCAACAACGTGTCCCTGACGCTGCCGGCGACGCCGTACCTG  60
                 ||||||||||||||||||||||||||||||||||||||||||||||||||||||||||||
Sbjct  21954299  GGGCGCTACAGGTGGTCCATCAACAACGTGTCCCTGACGCTGCCGGCGACGCCGTACCTG  21954358

Query  61        GGCGCCTTCCACCACGGCCTCCAGGACAGCGCGTTCGACGCGTCCGGCGAGCCGCCGGCG  120
                 ||||||||||||||||||||||||||||||||||||||||||||||||||||||||||||
Sbjct  21954359  GGCGCCTTCCACCACGGCCTCCAGGACAGCGCGTTCGACGCGTCCGGCGAGCCGCCGGCG  21954418

Query  121       GCGTTCCCGGAGGACTACGACGTGATGAGGCCGCCGGCGAACAACGCGACGACGGCGAGC  180
                 ||||||||||||||||||||||||||||||||||||||||||||||||||||||||||||
Sbjct  21954419  GCGTTCCCGGAGGACTACGACGTGATGAGGCCGCCGGCGAACAACGCGACGACGGCGAGC  21954478

Query  181       GACAGGGTGTTCCGGCTGCGACACGGCGGCGTGGTGGACGTGGTGCTCCAGAACGCCAAC  240
                 ||||||||||||||||||||||||||||||||||||||||||||||||||||||||||||
Sbjct  21954479  GACAGGGTGTTCCGGCTGCGACACGGCGGCGTGGTGGACGTGGTGCTCCAGAACGCCAAC  21954538

Query  241       ATGCTGAGGGAGGAGGTGAGCGAGACGCACCCGTGGCACCTCCACGGCCACGACTTCTGG  300
                 ||||||||||||||||||||||||||||||||||||||||||||||||||||||||||||
Sbjct  21954539  ATGCTGAGGGAGGAGGTGAGCGAGACGCACCCGTGGCACCTCCACGGCCACGACTTCTGG  21954598

Query  301       GTGCTCGGCTACGGCGACGGCCGGTACGACCCGGCGGCGCACGCGGCGGGGCTCAACGCC  360
                 ||||||||||||||||||||||||||||||||||||||||||||||||||||||||||||
Sbjct  21954599  GTGCTCGGCTACGGCGACGGCCGGTACGACCCGGCGGCGCACGCGGCGGGGCTCAACGCC  21954658

Query  361       GCCGACCCGCCGCTGCGGAACACGGCGGTGGTCTTCCCGCACGGGTGGACGGCGCTTCGG  420
                 ||||||||||||||||||||||||||||||||||||||||||||||||||||||||||||
Sbjct  21954659  GCCGACCCGCCGCTGCGGAACACGGCGGTGGTCTTCCCGCACGGGTGGACGGCGCTTCGG  21954718

Query  421       TTCGTCGCCAACAACACCGGCGCGTGGGCGTTCCACTGCCACATCGAGCCGCACCTCCAC  480
                 ||||||||||||||||||||||||||||||||||||||||||||||||||||||||||||
Sbjct  21954719  TTCGTCGCCAACAACACCGGCGCGTGGGCGTTCCACTGCCACATCGAGCCGCACCTCCAC  21954778

Query  481       ATGGGCATGGGCGTCGTCTTCGTCGAGGGGGAGGACAGGATGCACGAGCTCGACGTGCCC  540
                 ||||||||||||||||||||||||||||||||||||||||||||||||||||||||||||
Sbjct  21954779  ATGGGCATGGGCGTCGTCTTCGTCGAGGGGGAGGACAGGATGCACGAGCTCGACGTGCCC  21954838

Query  541       AAGGACGCCATGGCGTGCGGCCTCGTCGCCAGGACGGCCGCCACGCCGCTCACCCCGGCA  600
                 ||||||||||||||||||||||||||||||||||||||||||||||||||||||||||||
Sbjct  21954839  AAGGACGCCATGGCGTGCGGCCTCGTCGCCAGGACGGCCGCCACGCCGCTCACCCCGGCA  21954898

Query  601       ACGCCGCTGCCTCCGTCGCCGGCGCCGGCGCCATGA  636
                 ||||||||||||||||||||||||||||||||||||
Sbjct  21954899  ACGCCGCTGCCTCCGTCGCCGGCGCCGGCGCCATGA  21954934
```

**Figure S1.** Sequence alignment of the *REL1 interacting protein 5* (*RIP5*) obtained from the Y2H screening experiment with the genome of Nipponbare.

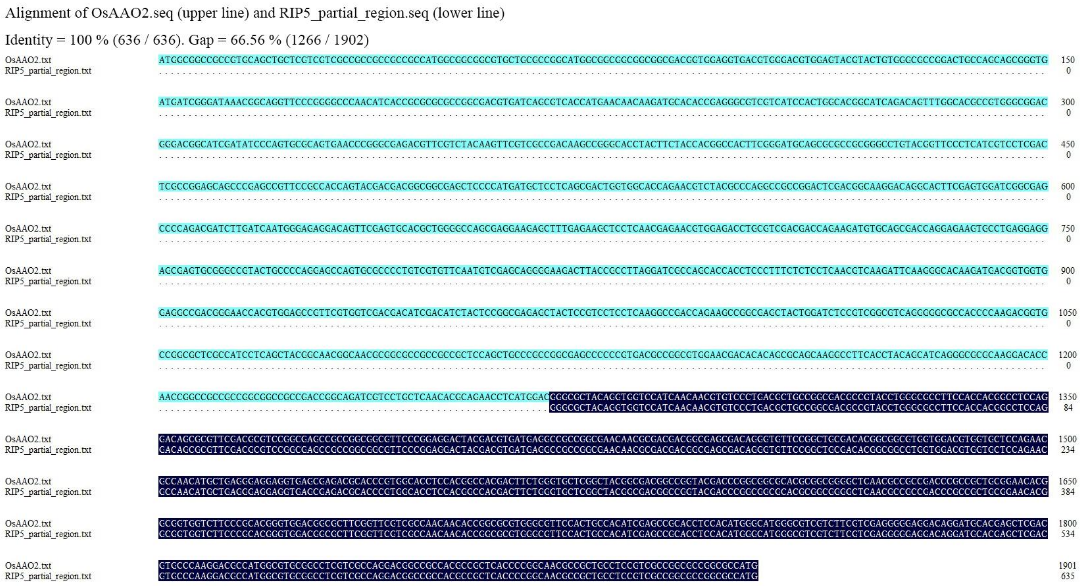


**Figure S2.** Sequence alignment of *OsAAO2* and *RIP5*_partial_region obtained from the Y2H screening experiment.

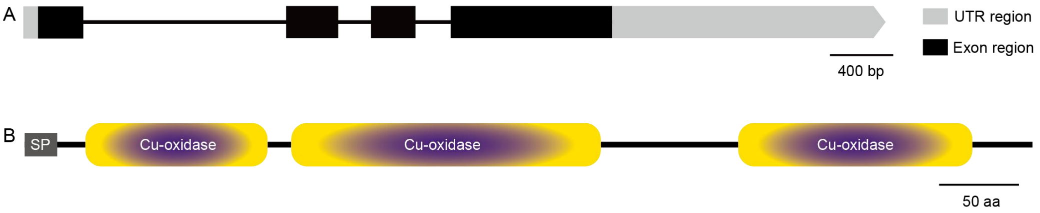


**Figure S3.** Gene and protein structure of RIP5. (A) Gene structure of *RIP5*. Exon region and UTR region are represented by black boxes and grey boxes. Scale bar, 400 bp. (B) Protein structure of RIP5. SP is signal peptide. Scale bar, 50 aa.

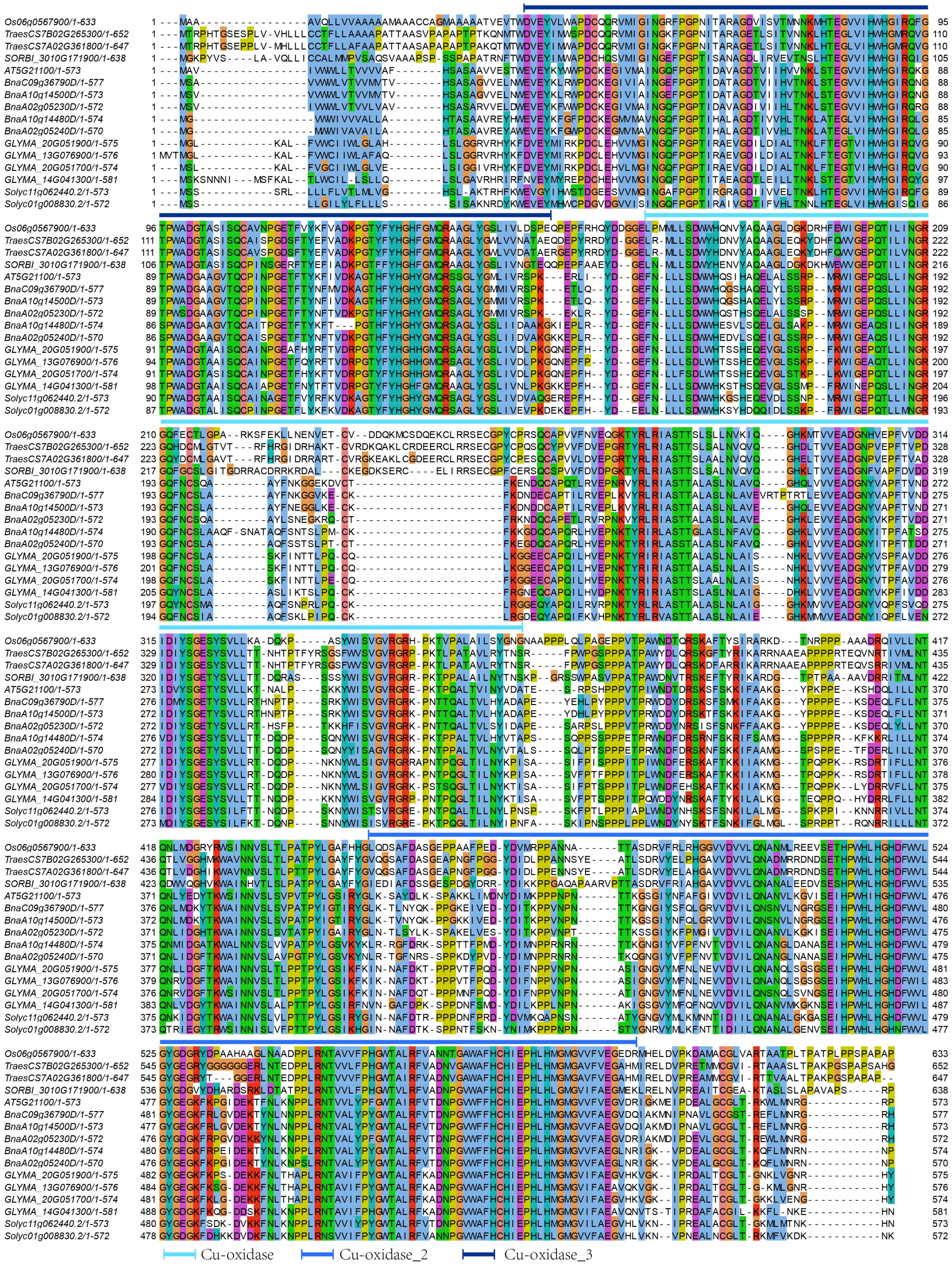


**Figure S4.** Multiple sequence alignment of the homologous genes of *RIP5* in arabidopsis, soybean, sorghum, maize, wheat, rape and tomato.

**Table S1**. List of primers used in this study.

| Application | Primer | Sequence（5'-3'） |
| --- | --- | --- |
| plasmid construction | AD-RIP5-F | GCCATGGAGGCCAGTGAATTCATGGCGG CCGCCGTGCAG |
| plasmid construction | AD-RIP5-R | CAGCTCGAGCTCGATGGATCCTCATGGCG CCGGCGCCGG |
| plasmid construction | RIP5-pSPYCE-F | TATCCAGATCCAGTGGGATCCATGGCGG CCGCCGTGCAG |
| plasmid construction | RIP5-pSPYCE-R | CGCACTAGTAAGCTTGGTACCTGGCGCCG GCGCCGGCGA |
| plasmid construction | RIP5-pRTVnGFP-F | GAGGACTTGAATGAAGGATCCATGGCGG CCGCCGTGCAG |
| plasmid construction | RIP5-pRTVnGFP-R | CGCACTAGTAAGCTTGGTACCTCATGGCG CCGGCGCCGG |
| plasmid construction | RIP5-pC-F | AACGATACTCGAGGGGGATCCATGGCGG CCGCCGTGCAG |
| plasmid construction | RIP5-pC-R | GGGAAATTCGCTAGTGGATCCTCATGGCG CCGGCGCCGG |
| Genotype analysis | V-Cas9-F | AGCCCTGCCTTCATACGCTA |
| Genotype analysis | V-Cas9-R | CTGCTGGGACTCCGTGGATA |
| Genotype analysis | P1 | GTGATGATCGGGATAAACGGC |
| Genotype analysis | P2 | GTTCATGGTGACGCTGATCACG |
| Genotype analysis | P3 | CGTGACACACACCACTCACT |
| Genotype analysis | P4 | TGTGTCCCATCCACCCAAAA |
| Genotype analysis | RIP5OE-F | AGGACCTCGACCTCAACACAAC |
| Genotype analysis | RIP5OE-R | ACGACGAGCAGCTGCACG |
| Genotype analysis | rel1-39208 | ATTAATCGGCAATAGGTCTC |
| Genotype analysis | TDNA7777 | TCGTGGAAAAAGAAGACGTTC |
| Genotype analysis | rel1-72833 | CGAGATAATGCAGGAGTACAA |
| qRT-PCR | actin-F | TCCAGCAGATGTGGATTGCCAAGG |
| qRT-PCR | actin-R | TCTGGTACCCTCATCAGGCATCTG |
| qRT-PCR | qRIP5-F | CCCATTCCAGTTAAATGCCATT |
| qRT-PCR | qRIP5-R | AAACCACTCTTCATCAGTGAGT |
| qRT-PCR | OsMYB2-F | GGGCTGAAACGCACAGGCAAGA |
| qRT-PCR | OsMYB2-R | CTGCTTGGCGTGCTTCTGC |
| qRT-PCR | POX22.3-F | CAGCTGCTCCAAGGTGAACTC |
| qRT-PCR | POX22.3-R | AGATTTGCTTCCAGCAACGAA |
| qRT-PCR | OsbHLH148-F | AGCTACGCCGACCTCTACG |

| Application | Primer | Sequence（5'-3'） |
| --- | --- | --- |
| qRT-PCR | OsbHLH148-R | GGAAGCTATTATTTGTATGATTGTA |
| qRT-PCR | OsDT11-F | TGTGTGACGCCGTGCAAT |
| qRT-PCR | OsDT11-R | CCAGGGCCGGAAGCTAGA |
| qRT-PCR | OsWRKY76-F | TTCCGAATGCTTTTCTGCTG |
| qRT-PCR | OsWRKY76-R | ATCGTGAGGCCCGATAGAAG |
| qRT-PCR | OsLEA3-F | CGGCAGCGTCCTCCAAC |
| qRT-PCR | OsLEA3-R | CGGTCATCCCCAGCGTG |

**Table S2.** Physical and chemical parameters of genes in the *OsAAOs* family

| Uniport_entry name | Gene ID | Name | Protein length (aa) | Molecular weight / kDa | Protein isoelectric point (pI) | Instability index | GRAVY | Subcellular localization |
|---|---|---|---|---|---|---|---|---|
| A0A0P0WY31_Q5Z645_ORYSJ | Os01g0816700 | OsAAO1 | 553 | 61.66 | 8.83 | 32.30 (stable) | -0.203 | Extracellular |
| A0A0P0WYB7_Q5Z5T3_ORYSJ | Os06g0567200 | OsAAO2 | 581 | 62.98 | 7.34 | 35.05 (stable) | -0.075 | Chloroplast |
| Q7XIF0_ORYSJ | Os06g0567900 | OsAAO3 | 633 | 69.12 | 5.90 | 43.86 (unstable) | -0.231 | Extracellular |
| A0A0P0XMD7_B8BEW5_ORYSJ | Os07g0119400 | OsAAO4 | 545 | 59.07 | 7.17 | 41.87 (unstable) | -0.150 | Chloroplast |
| A0A8J8XW93_Q0J0J1_ORYSJ | Os09g0365900 | OsAAO5 | 577 | 62.87 | 7.38 | 35.04 (stable) | -0.156 | Chloroplast |
| A0A0P0V9P6_Q942J6_ORYSJ | Os09g0507300 | OsAAO6 | 574 | 62.05 | 6.98 | 37.50 (stable) | -0.117 | Cell wall |

**Table S3.** List of sample sequencing data quality summary in this study.

| Sample | Raw_reads | Clean_reads | Clean_bases | Error_rate | Q20 | Q30 | GC_pct |
|---|---|---|---|---|---|---|---|
| rip5-ko_PEG_ 1 | 39322474 | 38392556 | 5.76 G | 0.02 | 98.22 | 94.82 | 51.98 |
| rip5-ko_PEG_ 2 | 48078672 | 46927924 | 7.04 G | 0.02 | 98.22 | 94.85 | 52.96 |
| rip5-ko_PEG_ 3 | 40686302 | 39714814 | 5.96 G | 0.02 | 98.25 | 94.86 | 52.61 |
| ZH11_PEG_1 | 49920428 | 49295096 | 7.39 G | 0.03 | 97.6 | 93.29 | 51.7 |
| ZH11_PEG_2 | 45815086 | 45267186 | 6.79 G | 0.03 | 97.69 | 93.5 | 52.48 |
| ZH11_PEG_3 | 47386120 | 46730102 | 7.01 G | 0.03 | 97.68 | 93.44 | 52.65 |
